# Injectable Therapies for Orofacial Myofascial Pain: A Rapid Review of Randomized Controlled Trials

**DOI:** 10.3390/jcm15135143

**Published:** 2026-07-01

**Authors:** Karolina Grzybowska-Kowalczyk, Izabella Chyży, Kamila Chęcińska, Wojciech Macek, Maja Kosińska, Maciej Chęciński, Amelia Hoppe, Julia Kasprzycka, Oliwia Jagiełło, Tomasz Horodniczy, Zuzanna Baniak, Maciej Sikora

**Affiliations:** 1 Uśmiech Family Dental Clinic, Nastrojowa 26, 91-496 Łódź, Poland; karolina.orto.gk@gmail.com; 2 National Medical Institute of the Ministry of the Interior and Administration, Wołoska 137, 02-507 Warsaw, Poland; ichyzy@gcm.pl (I.C.); maciej.checinski@pimmswia.gov.pl (M.C.); maciej.sikora@pimmswia.gov.pl (M.S.); 3 Department of Oral Surgery, Preventive Medicine Center, Komorowskiego 12, 30-106 Cracow, Poland; ld.wojciech.macek@wp.pl (W.M.); majaakosinska@gmail.com (M.K.); amelia.a.hoppe@gmail.com (A.H.); 4 Department of Maxillofacial Surgery, Hospital of the Ministry of the Interior and Administration, Wojska Polskiego 51, 25-375 Kielce, Poland; 5 Miodowa Clinic, Głowaccy Medical and Dental Practice Professional Partnership, Kiekrz, Miodowa 2, 62-090 Rokietnica, Poland; 6 Gabinety Lekarskie Dent-im M.B. Pawelczyk Spółka Jawna, Różana 13/1, 61-577 Poznań, Poland; oliwiajagiellodent@gmail.com; 7 Ortho.pl Dental Office, Buforowa 34, 52-131 Wrocław, Poland; tomasz.horodniczy2@gmail.com; 8 Stomatologia Centrum, Moniuszki 3/7, 32-020 Wieliczka, Poland; baniakzuzanna@gmail.com; 9 Department of Biochemistry and Medical Chemistry, Pomeranian Medical University, Powstańców Wielkopolskich 72, 70-111 Szczecin, Poland

**Keywords:** orofacial myofascial pain, intramuscular injections, masticatory muscles, trigger points, botulinum toxin, platelet-rich plasma

## Abstract

**Background/Objectives:** Orofacial myofascial pain (MFP) is one of the leading causes of chronic orofacial pain, often resulting in functional limitations and a compromised quality of life. Intramuscular injection therapies appear to be a promising alternative for patients resistant to conservative treatment. The objective of this rapid review was to synthesize evidence from randomized controlled trials evaluating intramuscular injectable therapies for orofacial myofascial pain. Specifically, the review aimed to compare the clinical effects of different injectable agents on pain intensity, mandibular function, and patient-reported outcomes, and to identify methodological limitations and research gaps within the current evidence base. **Methods:** A comprehensive search across five databases (ACM, BASE, Cochrane, PubMed, and Scopus) was conducted on March 15, 2026. Randomized controlled trials (RCTs) published between 2022 and 2026 that investigated the use of active injectable agents into the masticatory muscles for clinically diagnosed myofascial pain syndrome were included. Data regarding post-interventional pain intensity, masticatory function, mandibular range of motion, and safety were extracted to compare therapeutic efficacy across interventions. **Results:** A total of five RCTs met the inclusion criteria. Eligible studies evaluated intramuscular injections of botulinum toxin A, platelet-rich plasma (PRP), magnesium sulfate, and lidocaine, with sample sizes ranging from 30 to 180 participants. Across all interventions, consistent reductions in pain intensity and enhancements in masticatory function were observed. Furthermore, no major adverse events were reported. **Conclusions:** Intramuscular injectable therapies represent an emerging approach for reducing orofacial myofascial pain, particularly as a treatment for patients with persistent symptoms.

## 1. Introduction

### 1.1. Rationale

Orofacial myofascial pain (MFP) is one of the leading causes of chronic orofacial pain [1,2]. It is associated with functional limitations, including impaired mandibular mobility and reduced chewing efficiency, and may indirectly compromise health-related quality of life [3].

Conservative management of MFP typically includes patient education, physiotherapy, psychotherapy, occlusal splints, and systemic pharmacotherapy [4,5]. However, when conservative treatment proves insufficient or symptoms become persistent, more invasive approaches may be required. Among these, acupuncture, dry needling, and intramuscular injections are considered the least invasive. Accordingly, injectable therapies have practical clinical relevance, particularly in patients with chronic symptoms or resistance to standard conservative treatment [5,6].

**Figure 1:**
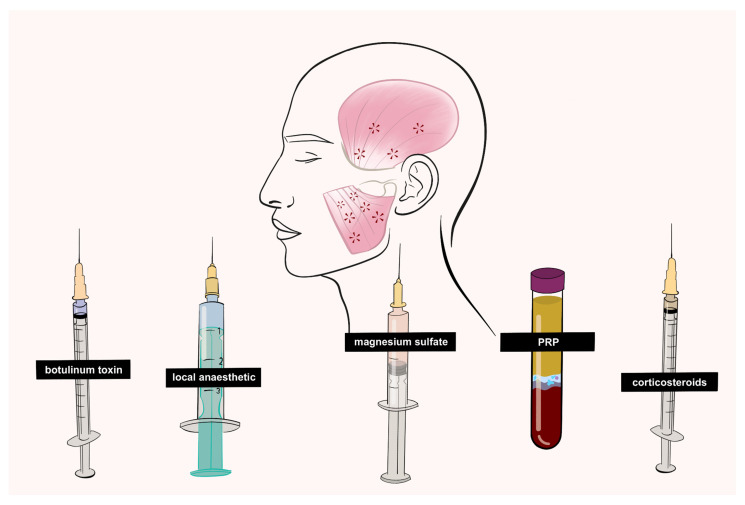
Schematic representation of intramuscular injection therapy for MFP. Schematic representation of intramuscular injection therapy for MFP. The illustration indicates trigger points in the temporalis and masseter muscles, as well as the primary substances reported in recent research: botulinum toxin, local anesthetics, magnesium sulfate, platelet-rich plasma (PRP), and corticosteroids. Original illustration created by the co-author (A.H) in Procreate v. 5.4 (Savage Interactive, 2026), for iPadOS.

Intramuscular injectable therapies for MFP comprise a heterogeneous group of interventions that are primarily classified according to the agent administered. These include local anesthetics, botulinum toxin, platelet-rich plasma, corticosteroids, collagen, and other emerging substances injected directly into the affected masticatory muscles (Figure 1) [7]. In recent years, such therapies have gained increasing attention as minimally invasive interventions for the management of orofacial myofascial pain, with the potential to improve both pain intensity and masticatory function [8].

Systematic reviews indicate that a variety of injectates, including botulinum toxin, platelet-rich plasma, corticosteroids, and local anesthetics, have been investigated, with several studies reporting clinically meaningful outcomes [6]. At the same time, substantial variability in treatment protocols, outcome measures, and follow-up durations limits direct comparison between studies and prevents firm conclusions regarding the relative efficacy of different agents [6,9]. Evidence from randomized controlled trials further suggests that biologic injectates such as platelet-rich plasma may provide superior outcomes compared with needling-based techniques, particularly over longer follow-up periods [10]. Nevertheless, despite these encouraging findings, the current evidence base remains limited and heterogeneous, and the role of individual injectable agents in MFP management has not yet been clearly established [6,9].

The available literature is characterized by marked heterogeneity in diagnostic criteria, injected substances, treatment protocols, comparison groups, and methods used to assess treatment outcomes [11,12]. As a consequence, clinicians are often required to make therapeutic decisions on the basis of scattered and methodologically diverse evidence. Moreover, the literature in this field is evolving rapidly, with new randomized clinical trials evaluating both established and novel injectable treatments for orofacial MFP [13]. Although previous review studies retain substantial scientific value, some may no longer fully reflect the current state of evidence [13].

In this context, a focused rapid review appears justified as a means of providing an up-to-date synthesis of the most recent randomized clinical evidence. Such an approach may better capture current clinical practice, contemporary injection protocols, and the present direction of therapeutic research while providing clinically relevant information for practitioners managing patients with orofacial myofascial pain [14].

### 1.2. Objectives

The objective of this rapid review was to synthesize evidence from randomized controlled trials investigating intramuscular injectable therapies for orofacial myofascial pain. The review sought to evaluate the effects of different injectable agents on pain intensity, mandibular function, and patient-reported outcomes, while also identifying methodological limitations and areas requiring further clinical investigation.

## 2. Materials and Methods

### 2.1. Protocol and Registration

This study was conducted as a rapid review of randomized controlled trials. The review followed a predefined protocol registered in the Open Science Framework (OSF) under registration number: osf.io/684zw and was designed to provide a focused and timely synthesis of contemporary evidence regarding intramuscular injectable therapies for orofacial myofascial pain. Given the rapid review approach, the search strategy was limited to selected databases and restricted to randomized controlled trials. The reporting of this review was informed by the PRISMA 2020 statement where applicable to rapid evidence synthesis [15].

### 2.2. Eligibility Criteria

The eligibility criteria were defined using the PICOTS framework [16]. We included randomized controlled trials involving human patients with clinically diagnosed myofascial pain syndrome affecting the masticatory muscles. Eligible interventions were active injectable agents administered intramuscularly into the masticatory muscles, including botulinum toxin, platelet-rich plasma, corticosteroids, local anesthetics, and hyaluronic acid. Studies investigating dry needling or acupuncture without injection, intra-articular temporomandibular joint injections, or systemic administration were excluded. Eligible comparators included placebo, sham injection, saline, needle insertion without an active agent, no treatment, waitlist, usual care, active comparators, and non-injectable comparators. To be eligible, studies had to report at least one prespecified outcome, namely pain intensity, masticatory function, or mandibular range of motion. We excluded non-randomized studies, observational studies, case series, case reports, reviews, and animal or in vitro studies. No language restrictions were applied, and non-English full texts were translated where feasible. The full eligibility criteria are summarized in Table 1.

### 2.3. Information Sources

The search covered studies published from 19 April 2021 through 15 March 2026, inclusive, thereby including studies published after the period synthesized by Nowak (now Szendera) et al. [6]. Searches were conducted in ACM, BASE, Cochrane, PubMed, and Scopus [17,18,19,20,21].

### 2.4. Search

Searches for primary research reports were conducted in the selected databases using a search strategy developed on the basis of the eligibility criteria. The strategy was refined during preliminary searches. The following query was ultimately applied across all databases: “(myofascial OR MPS) AND pain AND (trigger point* OR TrP) AND (injection* OR needl* OR punctur*) AND (intramuscular OR muscle*) AND (random* OR RCT)”.

### 2.5. Selection of Sources

All records identified through the database search were imported into the Rayyan screening software, and duplicates were removed by one of the authors (T.H.). The remaining records were screened in two stages using Rayyan (web application, https://www.rayyan.ai, accessed 15 March 2026; Rayyan Systems Inc., Cambridge, MA, USA). Before formal screening, the reviewers completed a calibration exercise to ensure consistent application of the eligibility criteria. First, titles and abstracts were independently reviewed by two authors (J.K. and O.J.) in a blinded manner to identify potentially relevant studies. Subsequently, the full texts of selected articles were independently assessed for eligibility against the predefined inclusion and exclusion criteria by the same two authors (J.K. and O.J.). Any disagreements were resolved through discussion and consensus. Reasons for exclusion at the full-text stage were recorded. The study selection process was documented using a PRISMA flow diagram [22].

### 2.6. Data Charting Process

Data from the included studies were charted using a standardized data charting form developed by the research team. The form was pilot tested on a small sample of studies and refined as necessary. Two reviewers (J.K. and O.J.) independently charted data from the selected articles using tables prepared in Google Sheets (web application, accessed from 15 March 2026 to 24 May 2026; Google LLC, Mountain View, CA, USA) to ensure accuracy and consistency. Any discrepancies were resolved through discussion and consensus between the reviewers.

### 2.7. Data Items

Data charting included, where reported, injection site, injected substance, volume per injection, number of injections, comparator, pain intensity, masticatory or functional outcomes, mandibular range of motion, adverse events and safety outcomes, and the main findings of each study. Additional information relevant to the objectives of the review was recorded when available.

### 2.8. Critical Appraisal of Individual Sources of Evidence

The included studies were critically appraised for methodological quality and potential sources of bias. The assessment was performed using the Joanna Briggs Institute critical appraisal checklist for randomized controlled trials [23].

### 2.9. Synthesis of Results

Given the heterogeneity in injectable agents, comparator groups, outcome measures, and assessment time points, the findings were synthesized narratively. The results were grouped according to the type of injectable intervention and summarized with particular attention to pain intensity, masticatory function, mandibular range of motion, and safety outcomes.

## 3. Results

### 3.1. Selection of Sources of Evidence

A systematic approach was employed to select sources of evidence for this rapid review. All identified records were imported into Rayyan, and duplicates were removed. Screening was performed in two stages: titles and abstracts were assessed first, followed by full-text review, as described above. Inter-rater agreement between reviewers was assessed using Cohen’s kappa coefficient, with substantial agreement observed at both the title/abstract stage (κ = 0.87) and the full-text stage (κ = 1.00). Any disagreements were resolved through discussion and consensus.

**Figure 2:**
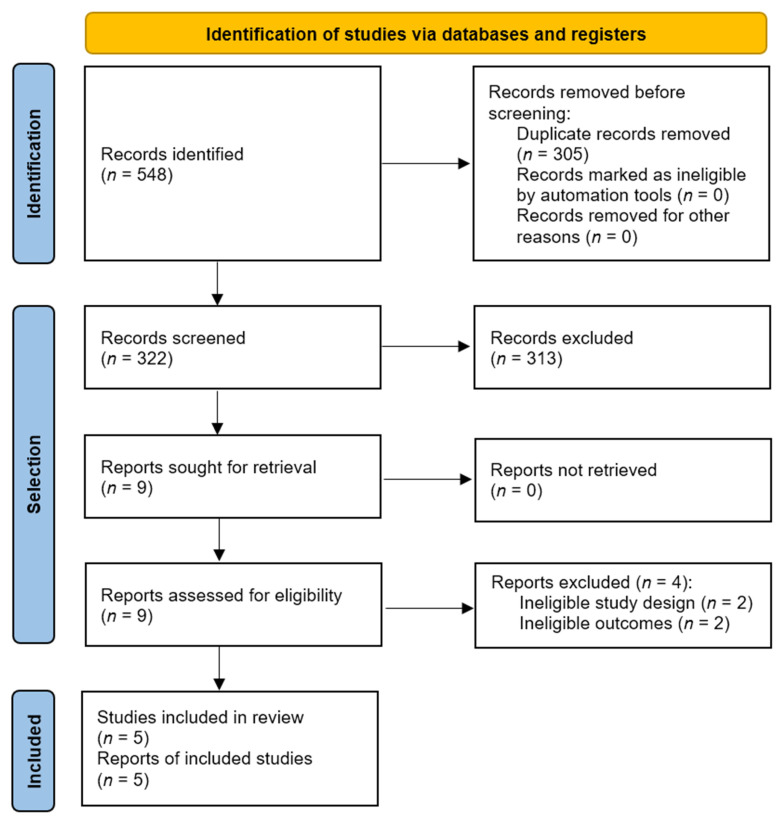
Flow diagram [22].

Reasons for exclusion at the full-text stage are summarized in Table A1. The study selection process is illustrated in Figure 2. The final set of included sources represents the evidence available to address the objectives of this review.

### 3.2. Characteristics of Sources Evidence

The search identified five randomized controlled trials that met the eligibility criteria. The included studies evaluated a range of injectable agents, including botulinum toxin, platelet-rich plasma, lidocaine, and magnesium sulfate. Considerable heterogeneity was observed across studies with respect to intervention protocols, comparator groups, outcome measures, and follow-up periods (Table 2).

All studies involved patients diagnosed with myofascial pain affecting the masticatory muscles, particularly the masseter muscle, with the presence of trigger points. One study additionally included a healthy control group. The sample sizes ranged from 30 to 180 participants.

All included studies evaluated interventions targeting the masseter muscle, with a focus on intramuscular or trigger point-based therapies. The investigated injectable interventions included botulinum toxin A, platelet-rich plasma (PRP), magnesium sulfate, and local anesthetics (lidocaine). Comparator groups varied across studies and included alternative injection techniques (intraoral vs. extraoral), pharmacological agents such as dexamethasone, non-injectable approaches such as dry needling and occlusal splint therapy, as well as combined treatment modalities.

Injection protocols differed substantially across studies (Table 3). One study administered a single dose of 100 units of botulinum toxin A and compared intraoral versus extraoral injection techniques. Two studies evaluated PRP injections (0.5 mL per trigger point), comparing them with dexamethasone and dry needling, respectively. One study investigated lidocaine trigger point injections, either alone or in combination with occlusal splint therapy. Another study assessed magnesium sulfate injections (2 mL per trigger point) compared with saline.

Primary outcomes assessed across studies included pain intensity, most commonly measured using the Visual Analogue Scale (VAS), and functional outcomes such as mandibular range of motion, reported as maximum mouth opening (MMO) or maximum interincisal opening (MIO). Additional outcomes included pressure pain sensitivity (PPI), quality of life (OHIP-14), patient satisfaction, sleep quality, and muscle stiffness assessed using shear wave elastography. Follow-up periods varied across studies, ranging from short-term (5 and 15 days) to medium-term follow-up of up to 6 months.

### 3.3. Critical Appraisal Within Sources of Evidence

Overall evidence was limited and mostly at high risk of bias, with one study at lower risk. This trial demonstrated stronger methodological rigor, including prospective design, trial registration, sample size calculation, and assessor blinding, although participant and operator blinding were not feasible. Therefore, the findings should be interpreted with caution due to the potential risk of bias. The results of the analysis are summarized in Table 4.

### 3.4. Results of Individual Studies and Synthesis of Evidence

The randomized controlled trial conducted by Shabaan et al. (2024) evaluated the effectiveness of intraoral versus extraoral administration of botulinum toxin A in patients with myofascial pain syndrome affecting the masseter muscle [24]. Both groups demonstrated a reduction in pain intensity (VAS) over time; however, the intraoral injection technique resulted in consistently lower pain scores at all follow-up points. Improvements in maximum mouth opening (MMO) were observed in both groups, with significantly greater improvement in the intraoral group at 1 and 3 months, but no significant difference at 6 months. Quality of life, assessed using the OHIP-14 questionnaire, improved in both groups, with significantly better outcomes in the intraoral group at 3 and 6 months. No significant adverse events were reported.

The randomized controlled trial conducted by Saba et al. (2025) compared platelet-rich plasma (PRP) and dexamethasone injections in patients with myofascial pain involving masseter muscle trigger points [25]. Both interventions resulted in significant improvements in pain intensity (VAS), pressure pain sensitivity (PPI), and mandibular range of motion (MIO) over time (*p* < 0.001). No statistically significant differences were observed between the groups. However, PRP showed a trend toward earlier pain reduction and complete resolution of tenderness at 1 month, while dexamethasone resulted in earlier improvement in mouth opening. No adverse events were reported.

Agarwal et al. (2022) conducted a randomized controlled trial comparing PRP injections with dry needling in patients with myofascial trigger points [10]. PRP demonstrated greater improvement in pain intensity and patient satisfaction, particularly at longer follow-up intervals, while both interventions resulted in improvements in mandibular functional movements. No major adverse events were reported.

The randomized controlled trial conducted by Saglam et al. (2024) evaluated lidocaine trigger point injections, occlusal splint therapy, and their combination in patients with myofascial pain related to temporomandibular disorders [26]. Significant improvements were observed across all treatment groups in pain intensity (VAS), maximum mouth opening (MMO), and masseter muscle stiffness at 1 and 3 months. However, no statistically significant differences were found between treatment modalities, indicating comparable clinical effectiveness. No adverse events were reported.

Refahee et al. (2022) assessed the clinical efficacy of magnesium sulfate injections compared with saline in patients with myofascial pain [27]. Magnesium sulfate resulted in significantly greater pain reduction and improved maximum mouth opening compared to saline, with sustained benefits observed up to 6 months. Quality of life (OHIP-14) also improved significantly in the magnesium sulfate group. No serious adverse events were reported; only transient local discomfort and redness were observed.

A summary of the outcomes and key findings of the included studies is presented in Table 5.

## 4. Discussion

### 4.1. Summary of Evidence

The available randomized evidence suggests that intramuscular injectable therapies can reduce pain in patients with orofacial myofascial pain, although functional outcomes are less consistent. Across the included studies, botulinum toxin A, platelet-rich plasma (PRP), magnesium sulfate, and local anesthetics were associated with clinical improvement, particularly in pain reduction [24,25,26,27,28].

Authors of the included trials reported pain reduction following botulinum toxin A, platelet-rich plasma, lidocaine, and magnesium sulfate injections, but the comparative effectiveness of individual agents remains unclear [24,25,26,27,28]. Shabaan et al. demonstrated greater improvement after intraoral botulinum toxin injection compared with the extraoral technique, suggesting that injection approach may influence outcomes [24]. Saba et al. found no significant difference between platelet-rich plasma and dexamethasone, whereas Agarwal et al. reported better long-term pain relief and patient satisfaction after platelet-rich plasma compared with dry needling [10,25]. Saglam et al. observed comparable improvements after lidocaine injections, occlusal splint therapy, and combined treatment, while Refahee et al. reported superior outcomes for magnesium sulfate compared with saline [26,27].

Beyond overall effectiveness, the included studies suggest that injectable agents may differ in the timing and durability of their effects. Botulinum toxin A may provide clinically relevant pain reduction, but its effect is temporary and may require repeated administration in clinical practice; in the included trial, some differences in functional improvement were no longer significant at 6 months [24,29]. In contrast, platelet-rich plasma showed a tendency toward more sustained pain relief in the study by Agarwal et al., although this was not confirmed in the comparison with dexamethasone reported by Saba et al. [10,25].

Comparable findings from other clinical contexts support the broader relevance of injectable therapies, while also showing that their effects depend on indication and protocol. Ye et al. reported improved outcomes after platelet-rich plasma administration in patients undergoing anterior cruciate ligament reconstruction, while Paget et al. described clinical benefits of platelet-rich plasma in ankle osteoarthritis [29,30]. For botulinum toxin, Rocha et al. observed significant pain reduction in hemiplegic shoulder pain, supporting its analgesic potential beyond the orofacial region [31]. These observations come from different clinical settings and should be interpreted with caution when applied to orofacial conditions.

Overall, heterogeneity in study design and intervention protocols limits direct comparisons and prevents firm conclusions regarding the relative effectiveness of individual agents.

### 4.2. Clinical Implications

Injectable therapies may be considered in patients with persistent orofacial myofascial pain, particularly when conservative treatment does not provide sufficient symptom relief. The available evidence suggests that different agents may have complementary clinical roles depending on symptom severity, expected speed of response, and treatment stage.

Botulinum toxin A may be useful when reduction in muscle activity is clinically relevant and relatively rapid pain relief is desired, although its temporary effect means that repeated administration may be needed to maintain the response [24,29]. Platelet-rich plasma may be considered when a longer-lasting biological effect is expected, as Agarwal et al. reported better long-term pain relief and patient satisfaction compared with dry needling [10]. However, because Saba et al. found no significant difference between platelet-rich plasma and dexamethasone, the current evidence does not support a firm preference for platelet-rich plasma over all active injectable comparators [25].

Magnesium sulfate may represent another potential option, as Refahee et al. reported greater pain reduction and improved maximum mouth opening compared with saline, with only transient local adverse effects [27]. Lidocaine injections may be useful in selected patients, particularly when a simpler analgesic intervention is considered, although Saglam et al. found outcomes comparable to occlusal splint therapy and combined treatment [26].

Taken together, these findings support an individualized rather than fixed treatment hierarchy. In clinical practice, it may be reasonable to consider botulinum toxin A when muscle hyperactivity is prominent, platelet-rich plasma when longer-term improvement is desired, magnesium sulfate as a potential alternative agent, and lidocaine when short-term analgesic support is the main therapeutic goal. This interpretation should remain cautious, because the available evidence is limited and direct comparisons between all agents are lacking. The clinical hypothesis is derived indirectly from the mechanism and limited data.

Each substance has a different mechanism of action. Botulinum toxin A reduces pain by blocking the release of acetylcholine at the neuromuscular junction and modulating nociceptive neurotransmitters, thereby reducing muscle hyperreactivity. Platelet-rich plasma, in turn, influences tissue repair by releasing numerous growth factors, which both support angiogenesis and reduce inflammation. Local anesthetics, such as lidocaine, induce muscle relaxation by blocking sodium channels and also have an analgesic effect. Magnesium sulfate exerts an analgesic effect by antagonizing NMDA receptors and stabilizing cell membranes. Corticosteroids inhibit the activity of proinflammatory cytokines, which consequently reduces local inflammation and muscle stiffness. These mechanisms are summarized in Table 6.

### 4.3. Safety Considerations

Adverse events were generally mild [27]. However, as noted by Pavicic et al., outcomes of botulinum toxin depend on technique and repeated administration [28], while variability in PRP protocols, highlighted in orthopedic studies, may influence both efficacy and safety [30,31].

### 4.4. Future Research Directions

Further randomized trials with larger sample sizes, standardized diagnostic criteria, and longer follow-up are needed to clarify the role of injectable therapies in orofacial myofascial pain. In particular, future studies should standardize intramuscular injection protocols, including the number of injections, injected volume, dose, timing between sessions, injection site, and follow-up duration.

Well-designed comparative trials are also needed. Placebo-controlled studies would help distinguish substance-specific effects from needling, placebo, or procedure-related effects, while head-to-head trials comparing botulinum toxin A, platelet-rich plasma, magnesium sulfate, corticosteroids, and local anesthetics would help define the relative clinical role of each agent.

Future research should also evaluate the temporal profile of treatment effects. This is particularly important because some agents, such as botulinum toxin A, may require repeated administration to maintain the effect, whereas platelet-rich plasma may have a different, potentially longer-term profile of action [27,29]. Standardized reporting of adverse events should also be included in future trials, as current safety data remain limited and inconsistently reported.

### 4.5. Strengths

This review provides a focused and up-to-date overview of intramuscular injectable therapies for orofacial myofascial pain and restricts inclusion to randomized controlled trials, thereby emphasizing higher-quality clinical evidence. A comprehensive search across multiple databases, with no language restrictions and translation of non-English full texts where feasible, reduced the risk of missing relevant studies. Study selection and data charting were performed independently by two reviewers, with discrepancies resolved by consensus, which enhanced the accuracy and reliability of the review process. In addition, the review focused on clinically meaningful outcomes, including pain intensity, masticatory function, and mandibular range of motion, which reflect both routine clinical practice and current directions in research on injectable therapies.

### 4.6. Limitations

Several limitations of the available evidence should be acknowledged. First, only five randomized controlled trials met the eligibility criteria, resulting in a relatively small evidence base. Second, substantial clinical and methodological heterogeneity was observed across studies, including differences in injectable substances, dosing protocols, comparator interventions, outcome measures, and follow-up durations. Third, several studies were characterized by methodological concerns, including small sample sizes, challenges related to blinding, and incomplete reporting of adverse events.

These limitations reduce confidence in direct comparisons between interventions and precluded quantitative synthesis of the available data.

## 5. Conclusions

The current evidence suggests that several intramuscular injectable therapies may provide clinical benefits for patients with orofacial myofascial pain. However, the available evidence is limited by the small number of randomized controlled trials and substantial methodological heterogeneity. No injectable agent demonstrated consistent superiority across studies.

Consequently, treatment decisions should be individualized and interpreted within the context of the limited evidence base.

Future well-designed randomized controlled trials employing standardized diagnostic criteria, treatment protocols, and outcome measures are needed to clarify the comparative effectiveness of available injectable therapies and to support evidence-based clinical decision-making.

## Figures and Tables

**Table 1:** Eligibility criteria.

	Criteria for Inclusion	Criteria for Exclusion
Population	Patients with myofascial pain syndrome affecting the masticatory muscles	Non-human populations; non-masticatory myofascial pain syndrome; no clear clinical diagnosis
Intervention	Active injectable agent administered intramuscularly into the masticatory muscle(s)	Dry needling; acupuncture without injection; intra-articular temporomandibular joint injection; systemic administration
Comparator	Placebo; sham injection; saline; needle insertion without active agent; no treatment; wait-list; usual care; active comparator; non-injectable comparator	No eligible comparator or control group
Outcome	At least one eligible outcome reported: pain intensity; masticatory function; mandibular range of motion	No relevant outcome reported
Timeframe	Studies published from 19 April 2021 through 15 March 2026	Studies published before 19 April 2021 or after 15 March 2026
Study design	Randomized controlled trials	Non-randomized studies; observational studies; case series; case reports; reviews; animal/in vitro studies
Language	No language restriction; non-English full texts translated where feasible	Full text unavailable and not translatable/assessable

**Table 2:** General characteristics of included studies.

First Author	Number of Patients	Population	Follow-Up
Shabaan [24]	42	Patients with myofascial pain (DC/TMD), masseter trigger points, and limited mouth opening	Baseline, 1, 3, and 6 months
Saba [25]	30	Patients with myofascial pain (masseter trigger points)	Baseline, day 5, day 15, 1 month
Agarwal [10]	30	Patients with myofascial trigger points in the masseter muscle diagnosed according to DC/TMD	Baseline, 2 weeks, 4 weeks, and 3 months; pain and patient satisfaction also assessed at 6 months
Saglam [26]	60	Patients with myofascial trigger points in the masseter muscle diagnosed according to DC/TMD	1 and 3 months
Refahee [27]	180	Patients with myofascial pain (DC/TMD) and masseter trigger points	Baseline, 1, 3, and 6 months

**Table 3:** Injection protocols across the included studies.

First Author	Injectable Agent	Injection Site	Route	Volume Per Injection	Number of Injections	Comparator
Shabaan [24]	Botulinum toxin A (Botox)	Masseter muscle trigger points	Intramuscular (intraoral vs. extraoral technique)	0.1 mL per trigger point	Single session (100 U total)	Extraoral injection technique
Saba [25]	Platelet-rich plasma (PRP) vs. dexamethasone	Masseter muscle trigger points	Intramuscular	PRP: 0.5 mL/TrP; dexamethasone: 0.4 mL/TrP	Not reported	Dexamethasone injection
Agarwal [10]	Platelet-rich plasma (PRP)	Masseter muscle trigger points	Intramuscular	0.5 mL per trigger point	Baseline treatment; repeat sessions allowed at follow-up if VAS reduction was <50%	Dry needling (DN)
Saglam [26]	Lidocaine	Masseter muscle trigger points	Masseter muscle trigger points	Not reported	Not reported	Occlusal splint; splint + injection; healthy controls
Refahee [27]	Magnesium sulfate (MgSO_4_)	Masseter muscle trigger points	Intramuscular	2 mL per trigger point	Single session (per trigger point)	Saline injection

**Table 4:** Risk of bias in the included studies.

First Author	Random Sequence Generation	Allocation Concealment	Blinding of Participants and Personnel	Blinding of Outcome Assessment	Incomplete Outcome Data	Selective Reporting	Other Sources of Bias	Overall
Shabaan [24]	Low	Unclear	High	Low	Low	Low	Moderate	High
Saba [25]	Low	High	High	High	Moderate	Low	High	High
Agarwal [10]	Low	Unclear	High	Low	Low	Low	High	High
Saglam [26]	Unclear	Unclear	High	High	Low	High	Moderate	High
Refahee [27]	Low	Low	Low	Low	Low	Low	Unclear	Low

**Table 5:** Results of the included studies.

First Author	Pain Intensity	Masticatory Function	MMO	Adverse Events	Key Findings
Shabaan [24]	Pain decreased in both groups; significantly lower in the intraoral group at all follow-ups	Not reported directly (OHIP-14 assessed quality of life instead)	Improved in both groups; significantly greater in the intraoral group at 1 and 3 months, with no significant difference at 6 months	Not reported	Intraoral botulinum toxin injection resulted in greater improvement in pain intensity, short-term MMO, and quality of life compared with the extraoral approach
Saba [25]	Significant reduction in VAS pain in both groups; no significant difference between groups	Not reported	Improved in both groups (MMO/MIO); no significant difference overall	Not reported	Both PRP and dexamethasone significantly improved outcomes, with no statistically significant differences between groups, although PRP showed a trend toward earlier pain reduction and dexamethasone toward earlier improvement in mouth opening
Agarwal [10]	Greater pain reduction in the PRP group; significant intergroup differences at 2 weeks, 4 weeks, and 6 months	Not reported directly	Mandibular functional movements improved in both groups; no significant intergroup differences reported for mouth-opening measures	No major adverse effects; transient post-procedural pain up to 24 h	PRP showed better pain relief and higher long-term patient satisfaction than dry needling
Saglam [26]	Occlusal splint; splint + injection; healthy controls	Not reported	Improved in all groups; no significant intergroup differences	Not reported	All treatment modalities demonstrated similar improvements in pain, MMO, and muscle stiffness, with no significant differences between groups
Refahee [27]	Significantly lower pain scores in MgSO_4_ group at all follow-ups	Not reported	Greater MMO in MgSO_4_ group up to 3 months; no significant difference at 6 months	Transient redness and mild discomfort resolving within 24 h	MgSO_4_ injections resulted in significantly greater pain reduction and improved quality of life compared to saline, with transient local adverse effects

**Table 6:** Summary of evidence on individual injectable agents.

Agent	Main Mechanism of Action	Main Effects	Observed Outcomes	Notes/Limitations
Botulinum toxin A	Blocks acetylcholine release at neuromuscular junction.	Muscle relaxation; reduction in hyperactivity and pain	Significant pain reduction (VAS), (Shabaan et al., 2025) [24]	Effect transient (3–6 months)
Platelet-rich plasma	Releasing PDGF, TGF-β, IGF-1, VEGF promotes myofiber regeneration, angiogenesis	Regenerative, anti-inflammatory, analgesia	Sustainable pain and function improvement vs. dry needling or dexamethasone (Agarwal 2022, Saba 2025) [10,25]	Protocols highly variable
Local anesthetics	Sodium-channel blockade	Analgesia, muscle relaxation.	Decreased pain and stiffness; similar outcomes vs. occlusal splint therapy (Saglam 2024) [26]	Transient effect; limited duration
Magnesium sulfate	NMDA-receptor antagonism; membrane stabilization	Central sensitization reduction	Greater pain relief and increased MMO vs. saline up to 3 months (Refahee 2022) [27]	Few studies available
Corticosteroids	Inhibition of prostaglandin and cytokine synthesis	Anti-inflammatory	Comparable to PRP in short-term pain relief (Saba 2025) [25]	Risk of tissue atrophy with repeated administration.

## Data Availability

Data is contained within the article.

## Abbreviations

DC/TMD	Diagnostic Criteria for Temporomandibular Disorders
DN	dry needling
JBI	Joanna Briggs Institute
MFP	myofascial pain
MIO	maximum interincisal opening
MMO	maximum mouth opening
MPS	myofascial pain syndrome
OHIP-14	Oral Health Impact Profile-14
OSF	Open Science Framework
PICOTS	Population, Intervention, Comparator, Outcome, Timeframe, Study design
PPI	pressure pain sensitivity
PRP	platelet-rich plasma
RCT	randomized controlled trial
TrP	trigger point
VAS	Visual Analogue Scale

## Appendix A

**Table A1:** Rejected reports.

Title	First Author, Publication Year	Identifier	Reason for Exclusion
Comparison of Lidocaine, Mepivacaine, and Dry Needling in Myofascial Pain Syndrome,	Münevveroğlu, 2025	NCT07079449	Excluded because only a trial registry record was available and no study results were reported
Comparative analysis of different dilution methods in botulinum toxin application	Develi, 2025	DOI: 10.1016/j.ijom.2025.04.1144	Excluded due to ineligible study design (retrospective study, not a randomized controlled trial)
Trigger Point Management	Shipton, 2023	PMID: 36791442	Excluded due to ineligible study design (clinical/narrative review, not a randomized controlled trial)
Comparative study of 5% dextrose vs. 18% dextrose prolotherapy in myofascial pain syndrome	Not reported, 2024	CENTRAL: CN-02775764	Excluded because only a trial registry record was available and no study results were reported

## References

[1] Reda B., Contardo L., Vidoni G., El-Outa A. (2024). Prevalence of Temporomandibular Disorders (TMD) in Dental Patients at a Specialized Regional Medical Center in Italy. Cureus.

[2] Cakir M., Ülker G.M.Y., Erdogan Ö. (2025). Prevalence and Comparison of Temporomandibular Disorders According to Axis I in RDC/TMD and DC/TMD: A Cross-Sectional Study. Quintessence Int..

[3] Riente A., Abeltino A., Serantoni C., De Giulio M.M., Bianchetti G., Santantonio M., Passali G.C., Capezzone S., Esposito R., De Spirito M. (2025). Using Quantitative Masticatory Dysfunction to Inform Pain Management in Trigeminal Neuralgia Through Electromyographic Monitoring. J. Oral Pathol. Med..

[4] Dąbkowska I., Sobiech L., Czępińska A., Bęben A., Turżańska K., Gawda P. (2025). Multimodal Approaches in the Management of Temporomandibular Disorders: A Narrative Review. J. Clin. Med..

[5] Dunning J., Butts R., Mourad F., Young I., Flannagan S., Perreault T. (2014). Dry Needling: A Literature Review with Implications for Clinical Practice Guidelines. Phys. Ther. Rev..

[6] Nowak Z., Chęciński M., Nitecka-Buchta A., Bulanda S., Ilczuk-Rypuła D., Postek-Stefańska L., Baron S. (2021). Intramuscular Injections and Dry Needling within Masticatory Muscles in Management of Myofascial Pain. Systematic Review of Clinical Trials. Int. J. Environ. Res. Public Health.

[7] Anwar N., Wei X., Jie Y., Zhao H., Jin H., Zhu Z. (2024). Current Advances in the Treatment of Myofascial Pain Syndrome with Trigger Point Injections: A Review. Medicine.

[8] Gupta P., Singh V., Sethi S., Kumar A. (2016). A Comparative Study of Trigger Point Therapy with Local Anaesthetic (0.5% Bupivacaine) Versus Combined Trigger Point Injection Therapy and Levosulpiride in the Management of Myofascial Pain Syndrome in the Orofacial Region. J. Maxillofac. Oral Surg..

[9] De La Torre Canales G., Câmara-Souza M.B., Ernberg M., Al-Moraissi E.A., Grigoriadis A., Poluha R.L., Christidis M., Jasim H., Lövgren A., Christidis N. (2024). Botulinum Toxin-A for the Treatment of Myogenous Temporomandibular Disorders: An Umbrella Review of Systematic Reviews. Drugs.

[10] Agarwal V., Gupta A., Singh H., Kamboj M., Popli H., Saroha S. (2022). Comparative Efficacy of Platelet-Rich Plasma and Dry Needling for Management of Trigger Points in Masseter Muscle in Myofascial Pain Syndrome Patients: A Randomized Controlled Trial. J. Oral Facial Pain Headache.

[11] Campana M.D., De Paolis G., Sammartino G., Bucci P., Aliberti A., Gasparro R. (2025). Cannabinoids: Therapeutic Perspectives for Management of Orofacial Pain, Oral Inflammation and Bone Healing—A Systematic Review. Int. J. Mol. Sci..

[12] Tardelli J.D.C., Gubitoso B., Botelho A.L., Valente M.L.D.C., Reis A.C.D. (2024). Efficacy of Acupuncture on Craniomandibular Myofascial Pain in Temporomandibular Disorder Patients: A Systematic Review. Heliyon.

[13] Steen J.P., Jaiswal K.S., Kumbhare D. (2025). Myofascial Pain Syndrome: An Update on Clinical Characteristics, Etiopathogenesis, Diagnosis, and Treatment. Muscle Nerve.

[14] Lubecka K., Chęcińska K., Bliźniak F., Chęciński M., Turosz N., Rąpalska I., Michcik A., Chlubek D., Sikora M. (2024). Update on Evidence and Directions in Temporomandibular Joint Injection Techniques: A Rapid Review of Primary Research. J. Clin. Med..

[15] Tricco A.C., Lillie E., Zarin W., O’Brien K.K., Colquhoun H., Levac D., Moher D., Peters M.D.J., Horsley T., Weeks L. (2018). PRISMA Extension for Scoping Reviews (PRISMA-ScR): Checklist and Explanation. Ann. Intern. Med..

[16] Amir-Behghadami M., Janati A. (2020). Population, Intervention, Comparison, Outcomes and Study (PICOS) Design as a Framework to Formulate Eligibility Criteria in Systematic Reviews. Emerg. Med. J..

[17] Association for Computing Machinery. https://www.acm.org/.

[18] BASE (Bielefeld Academic Search Engine): Wyszukiwanie Ogólne. https://www.base-search.net/.

[19] About the Cochrane Library|Cochrane Library. https://www.cochranelibrary.com/about/about-cochrane-library.

[20] PubMed. https://pubmed.ncbi.nlm.nih.gov/.

[21] Scopus|Abstract and Citation Database|Elsevier. https://www.elsevier.com/products/scopus.

[22] Page M.J., McKenzie J.E., Bossuyt P.M., Boutron I., Hoffmann T.C., Mulrow C.D., Shamseer L., Tetzlaff J.M., Akl E.A., Brennan S.E. (2021). The PRISMA 2020 Statement: An Updated Guideline for Reporting Systematic Reviews. BMJ.

[23] Barker T.H., Stone J.C., Sears K., Klugar M., Tufanaru C., Leonardi-Bee J., Aromataris E., Munn Z. (2023). The Revised JBI Critical Appraisal Tool for the Assessment of Risk of Bias for Randomized Controlled Trials. JBI Evid. Synth..

[24] Shabaan A.A., Kassem I., Aboulmagd I., Amer I.A., Shaaban A., Abd-El-Ghafour M., Refahee S.M. (2024). Effectiveness of Intra-Oral Botulinum Toxin Injection in Comparison to the Extra-Oral Approach on Pain and Quality of Life in Patients with Myofascial Pain: A Randomized Clinical Trial. Clin. Oral Investig..

[25] Saba Q., Rathore A., Yadav A., Pradhan S., Waikhom S., Uma A. (2025). Comparative Evaluation of the Efficacy of Platelet-Rich Plasma and Dexamethasone Injections in Myofascial Pain Trigger Points in the Masseter Muscle. J. Dent. Spec..

[26] Saglam R., Delilbasi C., Sayin Ozel G., Subasi I.D. (2024). Evaluation of the Effects of Occlusal Splint and Masseter Muscle Injection in Patients with Myofascial Pain: A Randomised Controlled Trial. J. Oral Facial Pain Headache.

[27] Refahee S.M., Mahrous A.I., Shabaan A.A. (2022). Clinical Efficacy of Magnesium Sulfate Injection in the Treatment of Masseter Muscle Trigger Points: A Randomized Clinical Study. BMC Oral Health.

[28] Pavicic T., Burgess C., Fabi S., Nestor M.S., Bee E.K., Imhof M., Dersch H., Sudimac V. (2025). Aesthetic Improvements Over Time: Long-Term Efficacy and Additional Outcomes of IncobotulinumtoxinA in the Simultaneous Treatment of Upper Facial Lines. J. Cosmet. Dermatol..

[29] Ye Z., Chen H., Qiao Y., Wu C., Cho E., Wu X., Li Z., Wu J., Lu S., Xie G. (2024). Intra-Articular Platelet-Rich Plasma Injection After Anterior Cruciate Ligament Reconstruction: A Randomized Clinical Trial. JAMA Netw. Open.

[30] Paget L.D.A., Reurink G., De Vos R.-J., Weir A., Moen M.H., Bierma-Zeinstra S.M.A., Stufkens S.A.S., Goedegebuure S., Krips R., Maas M. (2023). Platelet-Rich Plasma Injections for the Treatment of Ankle Osteoarthritis. Am. J. Sports Med..

[31] De Melo Carvalho Rocha E., Riberto M., Da Ponte Barbosa R., Geronimo R.M.P., Menezes-Junior M. (2023). Use of Botulinum Toxin as a Treatment of Hemiplegic Shoulder Pain Syndrome: A Randomized Trial. Toxins.

